# Rosuvastatin alleviates high-salt and cholesterol diet-induced cognitive impairment in rats via Nrf2–ARE pathway

**DOI:** 10.1080/13510002.2018.1492774

**Published:** 2018-07-01

**Authors:** Ibraheem Husain, Mohd Akhtar, Tushar Madaan, Malik Zainul Abdin, Mohammad Islamuddin, Abul Kalam Najmi

**Affiliations:** a Department of Pharmacology, School of Pharmaceutical Education and Research, Jamia Hamdard (Hamdard University), New Delhi, India; b Department of Biotechnology, School of Chemical and Life Sciences, Jamia Hamdard (Hamdard University), New Delhi, India

**Keywords:** Cognitive impairment, rosuvastatin, high-salt and cholesterol diet, oxidative stress, Nrf2, nuclear factor (erythroid-derived 2)-like 2

## Abstract

**Objective:** The objectives of our study were to investigate the possible effect of rosuvastatin in ameliorating high salt and cholesterol diet (HSCD)-induced cognitive impairment and to also investigate its possible action via the Nrf2-ARE pathway.

**Methods:***In silico* studies were performed to check the theoretical binding of rosuvastatin to the Nrf2 target. HSCD was used to induce cognitive impairment in rats and neurobehavioral studies were performed to evaluate the efficacy of rosuvastatin in enhancing cognition. Biochemical analyses were used to estimate changes in oxidative markers. Western blot and immunohistochemical analyses were done to check Nrf2 translocation. TUNEL and caspase 3 tests were performed to evaluate reversal of apoptosis by rosuvastatin.

**Results:** Rosuvastatin showed good theoretical affinity to Nrf2, significantly reversed changes in oxidative biomarkers which were induced by HSCD, and also improved the performance of rats in the neurobehavioral test. A rise in nuclear translocation of Nrf2 was revealed through immunohistochemical analysis and western blot. TUNEL staining and caspase 3 activity showed attenuation of apoptosis.

**Discussion:** We have investigated a novel mechanism of action for rosuvastatin (via the Nrf2–ARE pathway) and demonstrated that it has the potential to be used in the treatment of cognitive impairment.

## Introduction

Dementia is a neurological disorder that is associated with progressive loss of memory and difficulties in language, behavior, and cognition [1]. Approximately 47.47 million people were suffering from dementia worldwide in the year 2015 and it is estimated that this disease would affect 135.46 million people by the year 2050. An estimated $604 billion was spent on dementia care in the year 2010 and this figure is further estimated to rise to $1 trillion in the next decade [2,3]. Modern western food habits – especially those consisting of high salt and cholesterol diet (HSCD) – are one of the major risk factors for cognitive impairment. Such high-energy, high-saturated diets are commonly associated specifically with progressive deterioration in learning and memory [4]. Numerous longitudinal & cross-sectional correlational studies have concluded that increased consumption of food containing high levels of saturated lipids and fats leads to greater susceptibility for diseases such as dementia and have also indicated to cause impairment in memory retrieval speed as well as prospective memory [5]. In addition, high salt diets (and consequent hypertension) are also widely recognized to cause cognitive deficits by increasing hippocampal oxidative stress [6,7]. Therefore, metabolic syndrome caused by HSCD is a major risk factor for cognitive decline [8].

Apart from these, there are numerous other risk factors that are considered to have a role in the pathogenesis of dementia such as improper neurotransmitter modulation, neuroinflammation, oxidative stress, and mitochondrial dysfunction. Irregularities in cholinergic, GABAergic, glutamatergic, serotonergic, and histaminergic neurotransmission are commonly observed in Alzheimer’s disease – the most common form of dementia [9,10]. Additionally, there are several hypotheses that emphasize on the role of neuroinflammation in the pathophysiology of dementia. An abundance of human leukocyte antigen DR (HLA-DR) positive reactive microglia, for example, has been found in the brains of dementia patients in post mortem studies. The secretion of pro-inflammatory factors such as tumor necrosis factor and interleukin 1 and 6 because of activation of microglial cells by inflammatory signals leads to the formation of reactive oxygen species (ROS). This process is commonly observed in many neurodegenerative diseases such as Parkinson’s disease, Alzheimer’s disease, and fronto-temporal dementia [11]. A surge in the production of ROS, which in turn leads to oxidative stress, is another commonly observed phenomenon in both Alzheimer’s disease as well as vascular dementia (second most common form of dementia). There is a lot of evidence that reflects an increase in lipid peroxidation, DNA oxidation, and protein oxidation (and thus, oxidative stress) in patients of Alzheimer’s disease. Likewise, a diminution in the amount of antioxidants and rise in lipid peroxidation levels is commonly observed in vascular dementia [12]. The nuclear factor erythroid 2-related factor 2 (Nrf2)-antioxidant response element (ARE) pathway is a primary sensor and a master regulator of oxidative stress via its ability to modulate the expression of antioxidants and detoxifying genes [13]. Suppression of Nrf2–ARE pathway in animals has shown to make them more susceptible to a wide array of diseases including numerous cardiovascular, neurological, and metabolic disorders [14,15].

As mentioned earlier, diets containing high amounts of salt and cholesterol have been implicated to be a major risk factor in multiple neurodegenerative disorders. Apolipoprotein E (ApoE) is the cholesterol transporter in the body which is responsible for the maintenance of lipid and cholesterol homeostasis in the body. Studies have indicated a connection between the ApoE4 allele on the apolipoprotein gene with hypercholesterolemia and increased risk of development of Alzheimer’s disease [16]. Greater than 50% of subjects participating in clinical trials of Alzheimer’s disease are positive for ApoE4 [17]. Similarly, two meta-analyses have reported a correlation between statin therapy and a reduced risk in the development of Parkinson’s disease [18,19]. Rosuvastatin, a widely prescribed HMG-CoA reductase inhibitor, has been used for the treatment of dyslipidemia (LDL-C) and has been proven to be more effective in comparison to other members of its class [20]. A 6 week, randomized, open-label, parallel group, multi-center clinical trial called the STELLAR trial concluded that rosuvastatin was found to be overall more effective than atorvastatin, simvastatin, and pravastatin [21]. Rosuvastatin has also shown to attenuate ROS by various mechanisms such as inhibition of uncoupling of endothelial nitric oxide synthase, reduction of NADPH oxidase, upregulating enzymatic defense via antioxidants as well as protecting against DNA damage caused by hydrogen peroxide [22,23]. Because of the fact that both hyperlipidemia as well as oxidative stress is prominent risk factors in the pathogenesis of dementia, rosuvastatin – because of its dual action on both LDL-C and ROS – is an important prospective molecule for the treatment of cognitive impairment. Additionally, atorvastatin has also exhibited anti-oxidative, anti-nitrosative action, and neuroprotective role in preclinical studies [24,25]. Piracetam – a nootropic drug – was used as a positive control as it has proven activity in alleviating cognitive impairment [26].

In our previous studies, we found that rosuvastatin was able to inhibit AChE and amyloid beta peptide aggregation, as well as minimize the overexpression of NF-κB proteins – all of which have been found to play a crucial role in the pathology of multiple neurodegenerative disorders. This has indicated a potential role of rosuvastatin in ameliorating cognitive impairment [27,28]. In our present study, we intend to evaluate the efficacy of rosuvastatin in diminishing cognitive impairment induced by HSCD via its action on the Nrf2–ARE pathway.

## Materials and methods

### Drugs and chemicals

Rosuvastatin (RSV) and piracetam (PCT) were provided as a gift sample by Sun Pharmaceutical Industries Limited and Arbro Pharmaceuticals Limited (India), respectively. JC-1 (5, 5, 6, 6- tetrachloro-1,1,3,3- tetraethyl benzimidazolyl carbocyanine iodide) and ATP bioluminescence assay kit were procured from Sigma Aldrich (India). BCA protein assay kit was purchased from Span Diagnostics Limited, Gujarat, India. All other reagents used were of analytical grade. Double-distilled water was used throughout the experimental work.

### Animal procurement

The experimental protocol was approved by Institutional Animal Ethics Committee of Jamia Hamdard (Hamdard University), New Delhi, India (Registration no. JH/993/CPCSEA) as per the guidelines of Committee for the Purpose of Control and Supervision of Experiments on Animals. Female Wistar rats in the range of 150–200 gm of body weight were issued from Central Animal House Facility, Jamia Hamdard, New Delhi and housed in standard polypropylene cages (6 rats each cage) and had access to commercial standard pellet diet (Amrut rat feed, Nav Maharashtra Chakan Oil Mills Ltd, New Delhi, India). The rats were maintained under controlled room temperature (23 ± 2°C) and relative humidity (60 ± 5%) with 12 h light/12 h dark (day/night) cycle in the departmental animal house.

### Drug preparation

Suspensions of RSV and PCT were prepared by triturating the weighed amount of RSV (5, 10 & 15 mg/kg) and PCT (200 mg/kg) in 0.5% carboxy methyl cellulose (CMC) suspension (w/v) in normal saline, respectively [29,30]. High salt saline was prepared freshly by adding 2% w/v NaCl in water. Pellets of high cholesterol diet were prepared freshly by adding 1.25% cholesterol and 10% coconut oil in standard diet pellets and dried at room temperature.

### Molecular docking and MM-GBSA binding free energy

To predict binding modes of ligands to receptor on the basis of structures, molecular docking studies of the compounds were carried out on Maestro 10.5 program (Schrodinger Inc. USA). Three-dimensional structure of Nrf2-DNA complexes was retrieved from protein data bank (PDB code: 2FLU) to be used for the present docking study [31]. Molecular docking studies mainly involve selection and preparation of appropriate protein, grid generation, ligand preparation followed by docking & its analysis. The protein preparation was done in three steps, i.e. preprocess, review and modify, followed by refinement using ‘protein preparation wizard’ in Maestro 10.5. In these steps, water molecules were deleted and hydrogen atoms were added. Energy of the structure was minimized using OPLS 2005 force field. Similarly, ligands were prepared again using Force Field 2005. A default box was prepared by clicking the most appropriate site shown by running site map program. The ligand was docked into the grid generated from the protein using extra precision. The docking score, binding free energy and hydrogen bonds and pi–pi interaction formed with the surrounding amino acids are used to predict their binding affinities and proper alignment of these compounds at the active site of the Nrf2. The results were evaluated by docking score. Higher the docking score indicates more the binding affinity [32,33]**.**

Prime molecular mechanics-generalized born surface area (MM-GBSA) was calculated using Maestro 10.5. It is a tool to calculate ligand binding free energy. The test compound (rosuvastatin) along with the standard (piracetam) was used against the Nrf2-DNA complexes (PDB code: 2FLU). Protein preparation and the ligand preparation were done from the above described methods. Alternatively the MM-GBSA results may be procured running the MM-GBSA program directly from the file generated by running the docking protocol. The docking score, binding free energy and hydrogen bonds and pi–pi interaction formed with the surrounding amino acids are used to envisage their binding affinities and proper alignment of these compounds at the active site of the Nrf2-DNA [34].

### Experimental design

Prior to the commencement of experimental studies, animals were fed with standard rat food pellets for 2 days for acclimatization. The animals were randomly divided into six groups i.e. (Number of animals = 6 in each group). After that, animals were fed a HSCD *ad libitum* for 8 weeks to induce cognitive impairment [35]. After that, rats were treated with RSV (p.o.), PCT (i.p.) for 7 weeks in different doses (Table 1). The rats were observed for behavioral parameters and then immediately sacrificed for histopathological examination and estimation of biochemical parameters. The researchers were blinded to the treatment for behavioral tests.

**Table 1.** Different experimental groups and treatment conditions.

Group Treatment (*n* = 6)	Treatment
NC (Normal Control)	NS and ND^a^
TC (Toxic Control)	HSCD^a^
RSV5	HSCD^a^ and RSV (5 mg/kg b.wt)^b^
RSV10	HSCD^a^ and RSV (10 mg/kg b.wt)^b^
RSV15	HSCD^a^ and RSV (15 mg/kg b.wt)^b^
PCT200	HSCD^a^ and PCT (200 mg/kg b.wt)^b^

Note: *n*: number of rats in each group; HSCD: high salt and cholesterol diet; NS: normal saline; ND: normal diet; RSV: rosuvastatin; PCT: piracetam.

^a^Administered once daily from 1st to 105th day.

^b^Administered once daily from 57th to 105th day.

### Step-down type passive avoidance test

After treatment, the step-down type passive avoidance test was performed according to the previously described method [36]. At the beginning of training, rats were placed in the wooden platform and were allowed to adapt for 2 min. When the rats stepped down from the platform and placed all paws onto the grid floor, electric currents were delivered for 15 seconds. Then the rats would jump onto the platform to avoid the electric stroke, and the electric currents were maintained for 5 min. After a 24 h interval, the rats were again placed on the platform, and the latency to step down on the grid for the first time and the number of errors subjected to shocks within 3 minutes were measured as learning performances.

### Measurement of GSH, GPx, and MDA

Reduced glutathione (GSH) was estimated by a colorimetric method [37]. Equal amounts of brain homogenate (w/v) and 10% trichloroacetic acid were mixed and centrifuged at 3000 rpm for 15 minutes. 2 mL of phosphate buffer (pH 7.4), 0.5 mL 5,5-dithiobisnitro benzoic acid (DTNB) and 0.4 mL of double-distilled water were added to 0.01 mL of supernatant. Then, the mixture was vortexed and their absorbance was recorded at 412 nm within 15 minutes of the addition of DTNB.

Glutathione peroxidase (GPx) activity in brain was assayed spectrophotometrically through the glutathione/NADPH/GR system, by the dismutation of H_2_O_2_ at 340 nm [38,39]. In this assay, the enzyme activity is measured indirectly by means of NADPH disappearance. H_2_O_2_ is decomposed, generating oxidized glutathione (GSSG) from GSH. GSSG is regenerated back to GSH by the GR present in the assay media, at the expense of NADPH. The enzymatic activity was expressed in nmol NADPH min^−1^ mg^−1^ protein.

Malondialdehyde (MDA) is produced during lipid peroxidation and can be determined by the thiobarbituric acid reactive substances (TBARS) test [40,41]. 0.1 mL of brain homogenate was pipetted into a 13 × 100 mm test tube and incubated at 37°C in a metabolic shaker for 1 h. An equal volume of homogenate was pipetted into a centrifuge tube and placed at 0 °C and marked at 0 h incubation. After 1 h of incubation, 0.45 mL of 5% (w/v) chilled TCA and 0.45 mL 0.67% TBA were added and centrifuged at 4000×g for 10 min. Thereafter, supernatant was transferred to other test tubes and placed in a boiling water bath for 10 min. The absorbance of pink color produced was measured at 535 nm. The TBARS content was calculated by using a molar extinction coefficient of 1.56 × 105 M^−1^ cm^−1^ and expressed as nmol of TBARS formed/hr/mg of protein.

### Measurements of membrane potential, ROS, and ATP level

Preparation of Isolated Mitochondria: After behavioral test, the rats were euthanatized and brain mitochondria were isolated using a standard procedure [42]. First, brains were quickly removed and placed on ice. The brain of rats (*n* = 6) was carefully dissected following anatomical guidelines and placed in a glass Dounce homogenizer containing five times the volume of isolation buffer (215 mM mannitol, 75 mM sucrose, 0.1% BSA, 1 mM EGTA, 20 mM HEPES, pH 7.2). Following homogenization, a low-speed spin (1300 g for 5 min) to remove unbroken cells and nuclei was performed. The supernatant was carefully placed in fresh tubes, topped off with isolation buffer and spun down again at 13,000×g for 10 minutes. The supernatant was discarded and the resultant mitochondrial pellets were suspended in 500 µL of isolation buffer with 1 mM EGTA (215 mM mannitol, 75 mM sucrose, 0.1% BSA, 20 mM HEPES, pH 7.2) and 0.1% digitonin (in DMSO) was added to the pellets to disrupt the synaptosomes. After 5 minutes, samples were brought to a final volume of 2 mL using isolation buffer containing 1 mM EGTA and centrifuged at 13,000×g for 15 min. Next the pellets were resuspended in isolation buffer without EGTA (75 mM sucrose, 215 mM mannitol, 0.1% BSA, and 20 mM HEPES with the pH adjusted to 7.2 using KOH) and were centrifuged at 10,000×g for 10 min. The final mitochondrial pellet was suspended in isolation buffer without EGTA to yield a final protein concentration of approximately 10 mg mL^−1^ and immediately stored on ice. To normalize the results, the protein concentrations were determined with all the samples on the same micro well plate using a BCA protein assay kit.

For membrane potential measurements on isolated mitochondria, a 200 µM stock solution of JC-1 (5, 5, 6, 6- tetrachloro-1,1,3,3- tetraethyl benzimidazolyl carbocyanine iodide) was made using DMSO as the solvent. The assay buffer contained mitochondrial isolation buffer with the addition of 5 mM pyruvate and 5 mM malate. One hundred fifty microliter of assay buffer and 20 µL (1.2 mg mL^−1^ final concentration) of mitochondria were added to the wells of a 96-well black, clear bottom microplate (Corning) followed by the addition of 1 µM JC-1 and mixed gently. The microplate was covered with aluminum foil and left at room temperature for 20 minutes before reading. Fluorescence (excitation 530/25 nm, emission 590/35 nm) was then measured [43].

Production from isolated mitochondria, Mitochondrial ROS production was measured following incubation of isolated mitochondria with 25 µM 2,7- dichlorodihydrofluorescein diacetate for 20 min and then the DCF fluorescence (excitation filter 485/20 nm, emission filter 528/20 nm) was read as previously described [44]. In short, 100 μg (0.8 mg mL^−1^ final concentration) of isolated mitochondria were added to 120 µL of KCl-based respiration buffer with 5 mM pyruvate and 2.5 mM malate added as respiratory substrates and 25 µM 2,7-dichlorodihydrofluorescein diacetate. Mitochondrial ROS production in the presence of oligomycin (to increase ROS production) or FCCP (to decrease ROS production) was performed to ensure measurement values were within the range of the indicator.

ATP was determined luminometrically using ATP bioluminescence assay kit (Sigma, St. Louis, MO, USA) according to the provided protocol. Mitochondrial samples were assayed for ATP content using the ATP dependence of the light emitting luciferase-catalyzed oxidation of luciferin. ATP concentration was calculated according to a standard curve and related to protein content [45].

### Assay of caspase 3 activity

The assay of caspase-3 was performed according to the manufacturer’s instructions provided in the kit (Genxbio Health Sciences, Delhi, India) [46].

### Immunohistochemistry analysis of Nrf2 protein

For Immunohistochemistry analysis, paraffin sections of the brains were deparaffinized in xylene and then with acetone for 5 min each. Samples were rehydrated with a graded series of ethanol. After washing under running double-distilled water, antigen retrieval was performed by citrate buffer (pH 6). Three changes of section were done with tris-buffered saline (TBS) solution. These sections were then blocked with 1.5% normal goat serum for 1 h. Sections were then incubated with a purified goat polyclonal antibody raised against a peptide mapping at the N-terminus of Nrf2 of human origin (1:200; Santa Cruz Biotechnology) overnight at 4°C. Immunoreactivity was detected with biotinylated anti-goat rabbit secondary antibodies and the avidin-biotin-peroxidase complex. Immunoreactive signal was developed using diaminobenzidine as a substrate for 2 minutes. Photomicrographs were taken with a Meiji microscope enabled with lumenera camera. The images were analyzed with lumenera analyze 3 software. All immunohistochemical samples were analyzed in a blinded fashion [47].

### Terminal deoxyribonucleotidyltransferase (TdT)-mediated dUTP nick-end labeling (TUNEL) assay

TUNEL assay was used to detect DNA fragmentation by incorporating FITC–dUTP catalytically at the 3'-hydroxy end of the fragmented DNA according to the manufacturer’s instructions (Apo direct Kit, Roche). Single-cell suspensions from rat neural tissue were prepared [48]. Cell was washed twice with PBS. The cells were then finally resuspended in 0.2 mL of PBS and fixed by adding 1 ml of 2% paraformaldehyde on ice for 1 h, washed with PBS and incubated with 3% H2O2 in methanol for 10 min at 25 °C. This was followed by washing with PBS and the cells were then permeabilized with freshly prepared chilled 0.1% Triton X-100 for 5 min on ice. The cells were washed twice with PBS, after which 50 mL of reaction mixture containing TdT and FLUOS-labeled dUTP was added for 1 h at 37°C. The cells were washed and finally resuspended in PBS for data acquisition in a BD LSR II flow cytometer and analyzed using Deva software. Histogram analysis of FL-1H (*x*-axis; FITC fluorescence) was recorded to show the shift in fluorescence intensity compared with the control.

### Western blot

Homogenization of frozen brain samples was done with 15 volumes of (w/v) TBS buffer, which contained phosphatase and protease inhibitor cocktails after which the homogenate was centrifuged at 100,000 g at 4°C for 1 h. The pellet was resuspended in 15 volumes of 1% Triton X-100/TBS (TBSX) while the soluble fraction was collected as the TBS-soluble fraction. Following a 30 minute incubation at 0°C, centrifugation was performed for 1 h at 100,000 g at 4°C. The TBSX-soluble fraction (supernatant) was removed and the pellet was suspended in 70% formic acid (FA) followed by another centrifugation at 100,000 g at 4°C for 1 h. Neutralization of the FA fraction was performed with 20 volumes of 1 M Tris-bse (pH: 11) after which it was subsequently aliquoted and stored at –80°C. BCA proten assay kit (Bioworld, USA) to estimate total protein level of TBS-, TBSX-, and FA-. Equal quantities of protein extracts were subjected to SDS-PAGE for western blotting and transferred to polyvinylidene difluoride membranes (PVDF) membranes (Millipore, USA) [49]. The membranes were incubated at 4°C overnight with anti –Nrf2 (1:1000, Biolegend, USA) primary antibody after blocking in 5% non-fat milk for 1 hour at room temperature. Washing with TBST was performed and the membranes were then incubated with HRP-conjugated secondary antibodies at room temperature for a period of 2 hours, ECL kit (Bioworld, USA) was employed to visualize the signals and Image J software was used to determine band intensities.

### Statistical analysis

Results were expressed as the Mean ± Standard Error of Mean (SEM). The statistical significance of difference between groups was determined using one-way analysis of variance (ANOVA) followed by Tukey’s test. *P* value <.05was considered statistically significant. Error bars represent the SEM. All statistical tests were performed using the Prism software package (Version 4, GraphPad, San Diego, CA).

## Results

### Molecular docking and MM-GBSA binding free energy analysis

**Figure 1.**
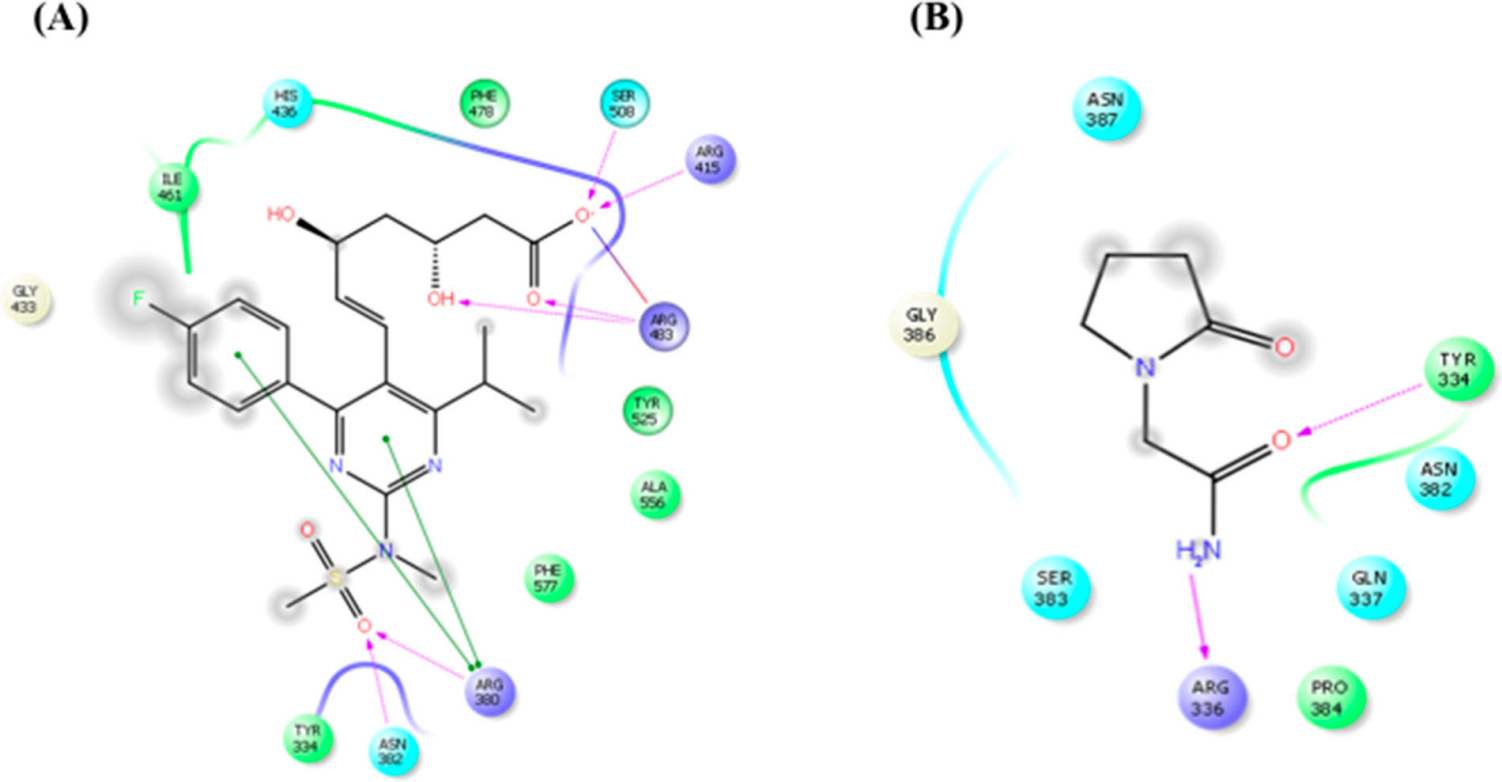
Molecular docking study of rosuvastatin and piracetam to Nrf2. (A) 2D ligand interaction representation of rosuvastatin showing hydrogen bond interaction with purple color arrow line and pi–pi stacking with green line in the binding site of nrf2. (B) 2D ligand interaction representation of piracetam showing hydrogen bond interaction with purple color arrow line and pi–pi stacking with green line in the binding site of nrf2.

The results were evaluated by interacting amino acids residues hydrogen bond, stacking amino acids residues pi-bond, docking score and binding free energy at the active sites of Nrf2-DNA. The docking scores determine the strength of interaction between a ligand and an enzyme (Figure 1). The lowest docking scores are the outcome of the best binding conformer at its receptor site or the active site of an enzyme. The docked ligand (PCT and RSV) displayed acceptable docking scores and binding free energy for Nrf2. These reports strongly suggest that the recognition of key structural features of RSV template will be helpful in designing and synthesizing new analogues with improved Nrf2 activity. All of these results are summarized in Table 2.

**Table 2.** Docking score and binding free energy of PCT and RSV with the active sites of Nrf2 along with the interacting amino acids.

Drug	Target (PDB ID)	Interacting amino acids residues H-bond	Stacking amino acids residues Pi-bond	Binding free energy (Kcal/mol)^a^	Docking score
Piracetam (PCT)	Nrf2 (2FLU)	TYR334, ARG336	ASN387, GLY386, SER383, PRO384, GLN337, ASN382	−15.317	−5.079
Rosuvastatin (RSV)		SER508, ARG415, ARG483, ARG380, ASN382	ILE461, HIS436, PHE478, TYR525, ALA556, PHE577, TYR334, GLY433	−20.637	−5.644

Note: PDB: Protein data bank.

^a^Binding free energy calculation using Prime MM-GBSA DG bind.

### Effect of RSV and PCT on latencies by step-down type passive avoidance test

**Figure 2.**
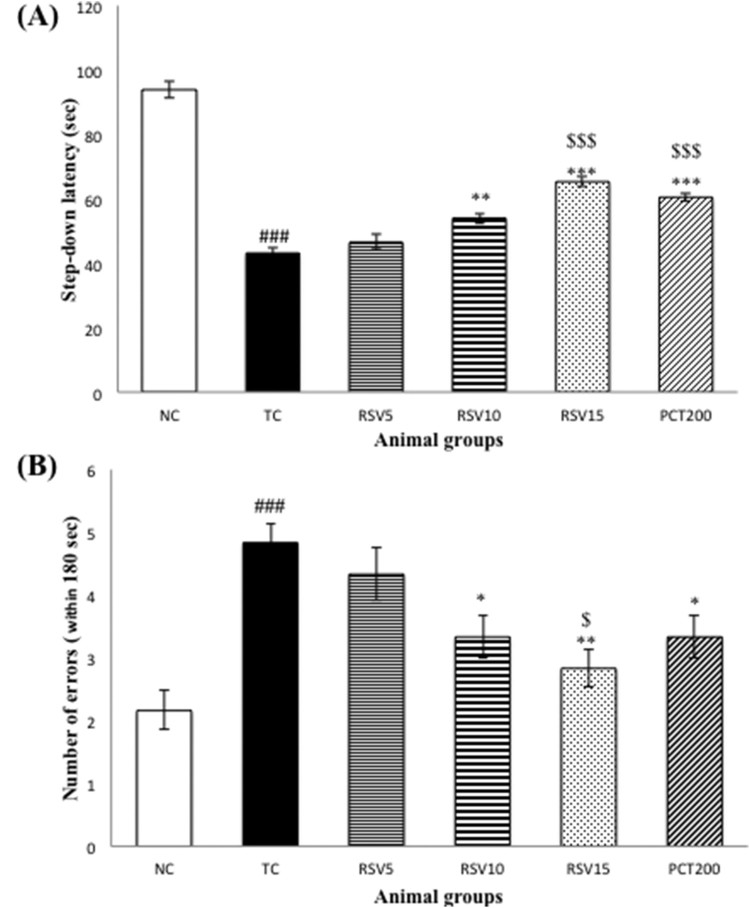
The step-down type passive avoidance test of rats with HSCD-induced cognitive impairment. (A) The average step-down latency. (B) The average number of errors within 3 min. Data are presented as mean ± SEM for six rats in each group. ^###^*p *< .001 vs. TC group. ***p < .001, **p < .01, *p < .05 vs. TC group. $$$*p *< .001, $*p *< .05 vs. RSV5 group.

The step-down type passive avoidance test is used to measure learning and memory in rodents. The step-down latencies and number of errors were observed for all animal groups. A decrease in the step-down latency reflects impairment in learning and memory, whereas an increase in the latency reflects normal cognition or alleviation from cognitive impairment. As far as the numbers of errors are concerned, an increase in the numbers of errors made implies impairment in cognition and a decrease in the number of errors reflects enhancement of memory. A significant decrease (^###^*p* < .001) in the step-down latency (Figure 2(a)) and increase in the number of errors (Figure 2(b)) was observed in the TC group (HSCD control group) when compared to the NC group. Non-significant (*p* > .05) changes were observed in the RSV5 group, however, RSV10 and RSV15 treated rats showed longer latency times and fewer error trials. Increase in latency time was more significant 15 mg RSV-treated group as compared to the 10 mg RSV-treated group (***p < .001 and **p < .01, respectively) with respect to TC group. The difference between RSV15 group and positive control (PCT200 group) was non-significant (*p* > .05).

### Effect on GSH, GPx, and MDA level

The assay for thiobarbituric acid reactive substances (TBARS) is one of the most commonly used methods to determine oxidative stress in humans as well as rodents. The TBARS assay ascertains the concentration of malondialdehyde generated as a result of unstable lipid peroxides [50]. A significant elevation in TBARS was observed in the toxic control group (HSCD control group) (^###^*p* < .001) as represented in Table 3. A dose dependent reduction in TBARS with increase in rosuvastatin dosage was noted. A significant decrease in TBARS was noted in RSV10 and RSV15 groups (*p < .05 and ***p < .001, respectively). 200 mg PCT was the most efficacious in reversing the increased MDA levels whereas a non-significant change was observed in R5 group.

**Table 3.** Effects of RSV, PCT and their combination on the activities of thiobarbituric acid reactive substance (TBARS), reduced glutathione (GSH) and glutathione peroxidase (GPx) in the brain tissue of experimental groups.

Animal groups	TBARS (nmol MDA/mg protein)	GSH (μmol GSH/mg protein)	GPx (nmol NADPH oxidized/min/mg protein)
NC	2.47 ± 0.07	8.21 ± 0.13	0.281 ± 0.005
TC	5.28 ± 0.15^###^	2.56 ± 0.09^###^	0.195 ± 0.007^###^
RSV5	4.19 ± 0.13	3.26 ± 0.12	0.209 ± 0.007
RSV10	3.711 ± 0.08*	3.80 ± 0.21**	0.251 ± 0.006***^‡‡‡^
RSV15	3.29 ± 0.09***^‡‡‡^	5.66 ± 0.32***^‡‡‡^	0.260 ± 0.005***^‡‡‡^
PCT200	3.23 ± 0.11***^‡‡‡^	6.00 ± 0.32***^‡‡‡^	0.258 ± 0.006***^‡‡‡^

Note: Data are presented as mean ± SEM for six rats in each group.

###*p* < .001 vs. TC group.

***p < .001.

**p < .01.

*p < .05 vs. TC group.

‡‡‡*p* < .001 vs. RSV5 group.

In addition to the TBARS assay, levels of reduced glutathione (GSH) and glutathione peroxidase (GPx) were also determined. GSH is one of the chief intracellular antioxidants and is produced as a result of the reduction of glutathione disulphide by nicotinamide adenine dinucleotide phosphate (NADPH) with glutathione reductase as the catalyst [51]. A significant reduction in GSH in HSCD-fed rats of TC group indicates the presence of oxidative stress. Rosuvastatin dose-dependently reversed the depletion in GSH. Reduction in GSH levels was significant in case of RSV10 and RSV15 groups (**p < .001 and ***p < .01, respectively). Similarly, a significant depletion in GPx levels was also observed in case of RSV10 and RSV15 groups (***p < .001 for both). The effect on RSV5 group was non-significant (*p* > .05) for both GSH and GPx whereas a significant reduction was observed in the PCT200 group in both cases (***p < .001). The difference between RSV15 group and PCT200 group was non-significant (*p* > .05).

### Effect on ROS production, mitochondrial ATP and mitochondrial membrane potential

**Figure 3.**
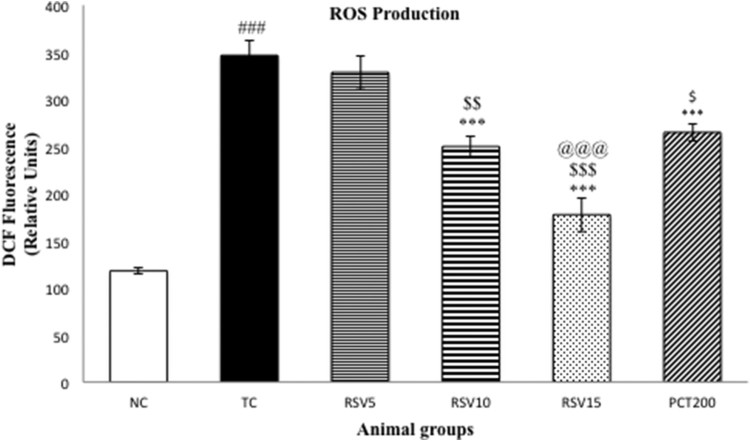
Effect of rosuvastatin and piracetam on ROS production. Data are presented as mean ± SEM for six rats in each group. ^###^*p *< .001 vs. TC group. ***p < .001, **p < .01, *p < .05 vs. TC group. $$$*p *< .001, $$*p *< .01, $*p *< .05 vs. RSV5 group. @@@*p *< .001 vs. PCT200 group.

In order to evaluate oxidative stress, ROS, as represented by Figure 3, were determined. A significant surge (^###^*p* < .001) in ROS was observed in the model groups. The efficacy of rosuvastatin in reducing ROS was inversely proportional to the administered dose. A non-significant change in ROS production was found in case of RSV5 group which was administered 5 mg of rosuvastatin, whereas significant diminution (***p < .001) in ROS was observed in the RSV10, RSV15, and PCT200 groups. However, 15 mg of RSV was found to be significantly more efficacious in reducing ROS when compared to 200 mg of piracetam (^$$$^*p* < .001).

**Figure 4.**
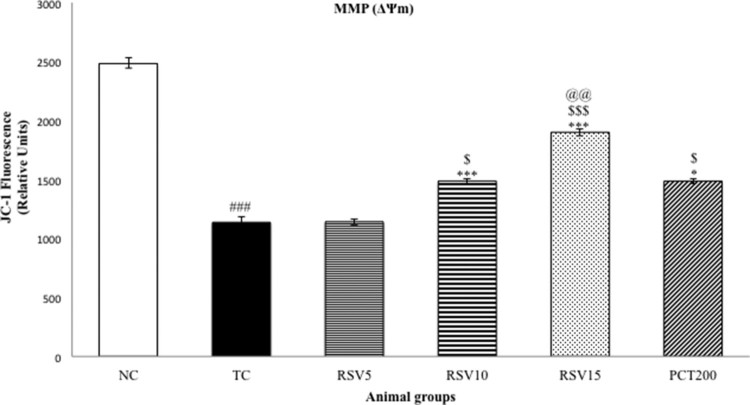
Effect of rosuvastatin and piracetam on MMP. Data are presented as mean ± SEM for six rats in each group. ^###^*p *< .001 vs. TC group. ***p < .001, **p < .01, *p < .05 vs. TC group. $$$*p *< .001, $$*p *< .01, $*p *< .05 vs. RSV5 group. @@*p *< .01, vs. PCT200 group.

**Figure 5.**
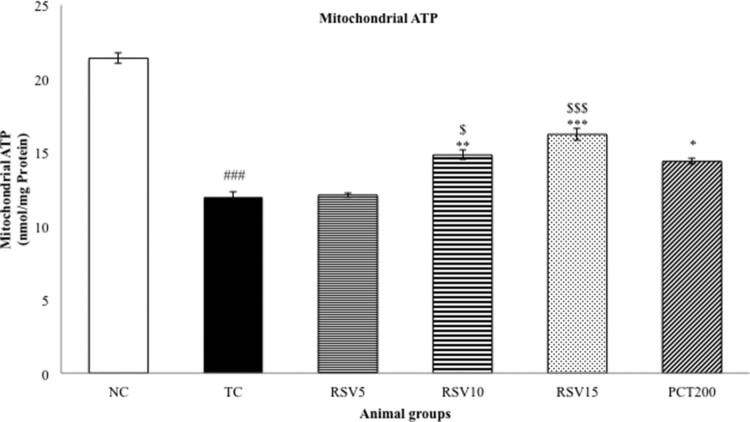
Effect of rosuvastatin and piracetam on mitochondrial ATP. Data are presented as mean ± SEM for six rats in each group. ^###^*p *< .001 vs. TC group. ***p < .001, **p < .01, *p < .05 vs. TC group. $$$*p *< .001, $*p *< .05 vs. RSV5 group.

Mitochondrial ATP and mitochondrial membrane potential (MMP) were determined as a measure of oxidative stress. A significant diminution in MMP and Mitochondrial ATP were observed in the TC group (^###^*p* < .001) as represented in Figures 4 and 5, respectively. The effect of 5 mg of rosuvastatin (in RSV5 group) was non-significant (*p* > .05) in reversing the reduced MMP and Mitochondrial ATP level. However, a significant reversal in the reduced MMP was observed in the RSV10, RSV15 and PCT200 groups (*p < .05 for RSV10 and PCT200, whereas ***p < .001 for RSV15). Hence, the efficacy of RSV15 in increasing the depleted MMP levels was more significant than that of PCT200. A similar trend was observed in case of mitochondrial ATP with rosuvastatin dose-dependently increasing the reduced levels. In this case, a non-significant difference between RSV15 and PCT200 groups was observed (*p* > .05).

### Effect on caspase 3 activity

**Figure 6.**
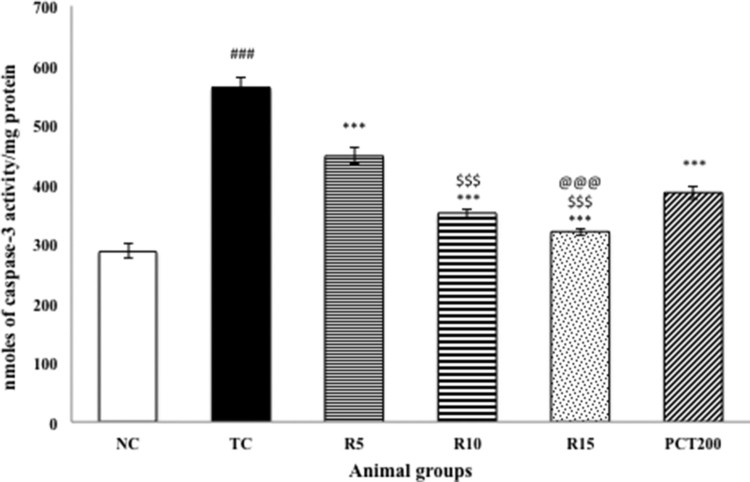
Effect of rosuvastatin and piracetam on caspase-3 activity. Data are presented as mean ± SEM for six rats in each group. ^###^*p *< .001 vs. TC group. ***p < .001, **p < .01, *p < .05 vs. TC group. $$$*p *< .001, $$*p *< .01, $*p *< .05 vs. RSV5 group. @@*p *< .01 vs. PCT200 group.

Figure 6 shows the effect of RSV on the brain activity of caspase-3. The HSCD-treated group showed significantly (*p* < .001) increased expression of activated caspase-3 in brain cells of rats as compared to normal control rats. Treatment with RSV (5, 10 and15 mg kg^−1^) in HSCD-treated animals significantly (*p* < .001) decreased the levels of activated caspase-3 as compared with the HSCD-treated group.

### Effect on brain immunohistochemistry analysis of nrf2 protein

**Figure 7.**
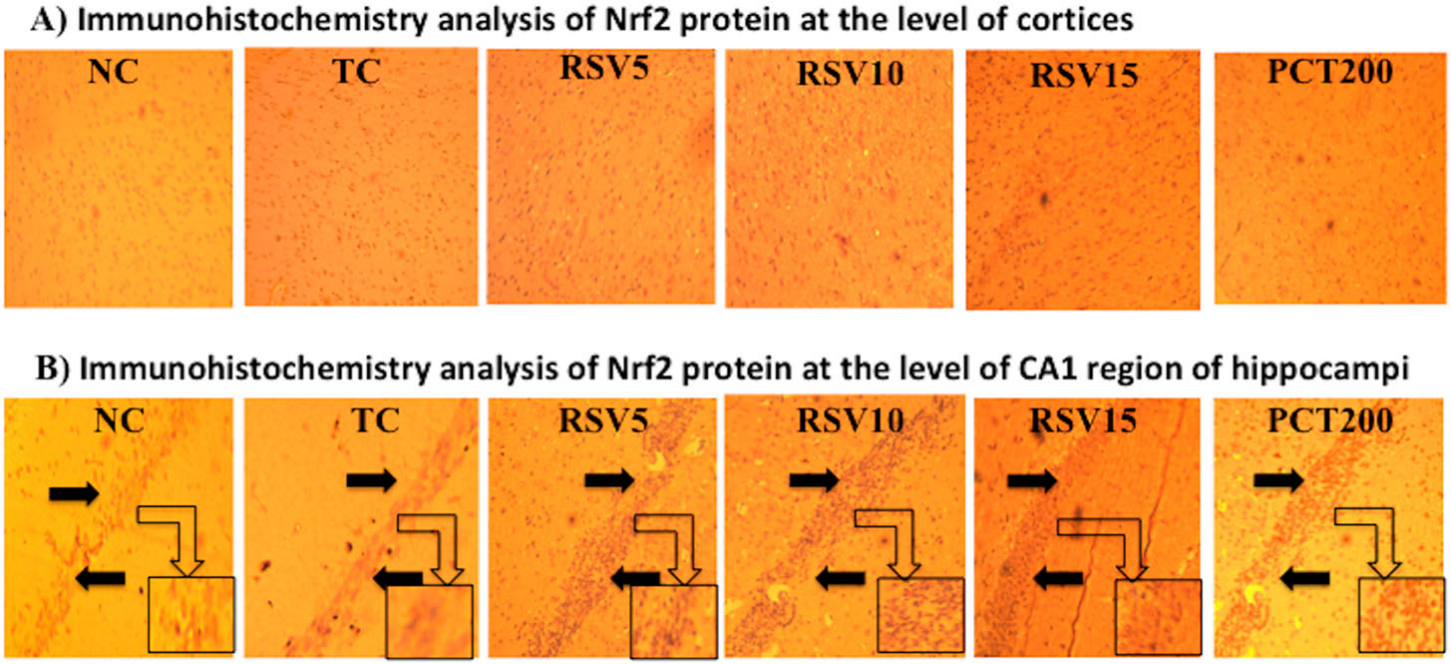
Immunohistochemistry analysis of Nrf2 protein by fluorescent microscope in coronal brain sections at 10x magnification. (A) Schematic illustration of cortices in groups by immunohistochemical staining for Nrf2. The profound expression of re Nrf2 were observed in TC group as compared to NC group, treatment groups of rosuvastatin and piracetam have shown effect on staining of nrf2. (B) Immunohistochemical staining for Nrf2 at the level of CA1 region of hippocampus. The profound expression of nuclear Nrf2 were observed in TC group as compared to NC group, treatment groups of rosuvastatin and piracetam have shown effect on staining of nrf2. Black arrows are showing the positively stained cells.

Further assessing the oxidative stress effects of HSCD on the brain of rats, we examined the expression of Nrf2 in cortex tissue and CA1 region of hippocampus. Moreover, Nrf2 proteins were mostly located in cellular plasma, and the positive cells had been stained dark brown. Low levels of constitutive expressions of Nrf2 were observed in normal cortex tissues and CA1 region of hippocampus in normal control (NC) group. After 15 weeks of HSCD, the immunoactivity of Nrf2 in cortex and CA1 region of hippocampus were significantly enhanced, and the positive cells of Nrf2 were markedly increased, with strong positive staining of Nrf2 both in cytoplasm and nucleus. Rosuvastatin treatment increased the nuclear expression of Nrf2 dose dependently in HSCD-fed rats (Figure 7).

### Assessment of DNA fragmentation by TUNEL assay

**Figure 8.**
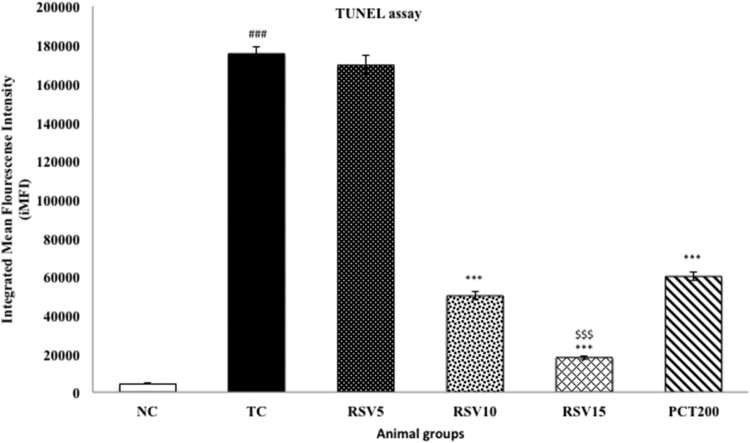
Effect of rosuvastatin and piracetam on DNA Fragmentation by TUNEL assay. Data are presented as mean ± SEM for six rats in each group. ^###^*p *< .001 vs. TC group. ***p < .001 vs. TC group. $$$*p *< .001 vs. RSV5 group.

Apoptosis is characterized by rapid DNA fragmentation, which can be quantified using flow cytometry by TUNEL assay [52]. HSCD resulted in nuclear DNA fragmentation as evidenced by an increase in dUTP-FLOUS labeling. In toxic control group, integrated mean fluorescence intensity (iMFI) significantly increased to 175348 (*p* < .001), respectively, in comparison with normal control (iMFI = 4422), respectively, providing strong evidence for apoptosis in HSCD-fed rats. RSV5 treated group exhibited a non-significant increase in iMFI (iMFI = 169387, *p* > .05). RSV10, RSV15 and PCT200 treated groups showed significantly decrease in iMFI (iMFI = 49912, 17902, 59939; *p* < .001), respectively, in comparison with toxic control group (Figure 8).

### Effect on activation of Nrf2 protein expression by western blot analysis

**Figure 9.**
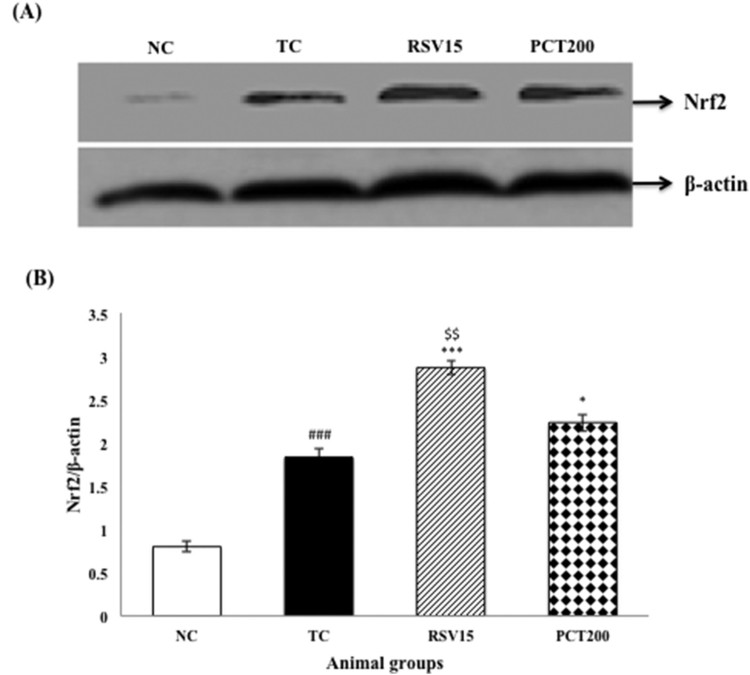
Effects of RSV and PCT on brain Nrf2 expression. (A) Western blot analysis of Nrf2. (B) Densitometry values for Nrf2 was normalized to β-actin and expressed as fold change relative to the normal control group. Data are shown as mean ± SEM (*n* = 3 rats per group). ^###^*p *<* *.001 vs. TC group. ***p < .001, **p < .01, *p < .05 vs. TC group. $$*p *< .01 vs. PCT200 group.

In our findings, the protein expression of Nrf2 in nuclear fractions was detected to gradually increase when treated with RSV & PCT when compared to the TC group, indicating the translocation of Nrf2 to nucleus (Figure 9(A,B)).

## Discussion

Classical pharmaceutical research was based on the ‘one molecule – one target’ approach. However, recent trends have shown that forthcoming research would be based on the ‘one molecule – multiple target’ paradigm. This has led researchers to not only design drugs that act on multiple targets at the same time, but also to evaluate ulterior targets and actions of presently available drugs. In our current study, we have tried to investigate the potential of rosuvastatin in the treatment of cognitive impairment induced by HSCD.

The nuclear factor erythroid 2-related factor 2 (Nrf2) is an endogenous, basic region leucine-zipper transcription factor that helps in the regulation of ROS and has anti-oxidative and cytoprotective actions. Under normal-physiological conditions, Nrf2 signaling is repressed by Keap1 and mainly sequestered in the cytoplasm. However, Nrf2 could be activated by oxidative stress, and then translocated into the nucleus to perform tasks. Nrf2 works by binding to the antioxidant response element (ARE) and consequently regulating various genes which are responsible for anti-oxidative, anti-inflammatory and mitochondrial protection actions. Coenzyme Q10, superoxide dismutase, quinone oxidorectuse, and glutathione-S-transferase, heme oxygenase-1 are some of the ARE-containing gene promoters [53,54]. These protective genes are activated in response to Nrf2 which ultimately leads to maintenance of redox balance in the body. They also aid in the elimination of damaged proteins, which are produced when the body is under xenobiotic or oxidative stress. Nrf2 is inhibited in various neurodegenerative disorders, including Alzheimer’s disease, Parkinson’s disease, amyotrophic lateral sclerosis, etc. Moreover, increase in expression of Nrf2 has shown to protect against the progression of diseases in murine models of the aforementioned disorders. Nrf2 has also shown protective action in mammalian cells against toxicity induced my amyloid-β-42 (Aβ42) peptide [55]. Therefore, Nrf2 serves as a potential target for the treatment of neurodegenerative diseases.

Our study began by performing *in silico* experiments to evaluate the theoretical affinity of RSV towards Nrf2 (2FLU) target. RSV formed five hydrogen bonds (SER508, ARG415, ARG483, ARG380, ASN382) with interacting amino acid residues, whereas PCT only made two (TYR334, ARG336). Similarly, RSV formed eight pi-bonds (ILE461, HIS436, PHE478, TYR525, ALA556, PHE577, TYR334, GLY433) with stacking amino acid residues, while PCT formed six (ASN387, GLY386, SER383, PRO384, GLN337, ASN382). Furthermore, the free binding energy and docking score of RSV were less than that of PCT, which indicates that RSV has better affinity than the positive control (PCT).

The passive avoidance paradigm is a popular behavioral test to evaluate learning, memory, and cognitive impairment in rodents as well as to identify compounds that alter cognitive processes [56]. We performed the step-down passive avoidance test to evaluate the efficacy of rosuvastatin in ameliorating cognitive impairment induced by high salt and cholesterol diet. The step-down latency was significantly less in the TC group as compared to the NC group which clearly indicates impairment of learning and memory in the TC group and is consistent with previous findings [57]. The latency increased linearly with increase in RSV dose which indicates that RSV ameliorated HSCD-induced cognitive impairment in a dose-dependent manner. The performance of the PCT200 group was intermediate between the R10 and the R15 groups whereas the longest step-down latency (after the NC group) was observed in the R10 + PCT200 group. A similar trend was observed when the numbers of errors of each group in 180 s were compared.

The generation of free radicals such as ROS and reactive nitrogen species which consequently leads to oxidative stress is a commonly accepted mechanism for various diseases and has been widely studied by researchers. There are several antioxidant systems in our body to counter the generation of free radicals. Some of these include antioxidant enzymes such as glutathione peroxidase and superoxide dismutase, antioxidant molecules like GSH, vitamin E, etc., and antioxidant proteins such as peroxyredoxin and thioredoxin. Our brain is particularly susceptible to damage caused due to oxidative stress and free radicals including damage caused to oxidation of polyunsaturated fatty acids (PUFA) which are present in high quantities in modern western diet, ultimately leading to damaged DNA, lipids, and proteins [58]. It is well established that increased levels of cholesterol and sodium in the body lead to a surge in the production of ROS and hence, cause oxidative stress which consequently leads to a multitude of metabolic syndrome – associated diseases including several neurological disorders [59,60]. A significant surge in ROS and TBARS and a significant reduction in GSH and GPx were observed in rats fed with HSCD. Rosuvastatin administration, particularly the 10 and 15 mg doses, were able to significantly minimize the increase in TBARS and ROS, and also significantly ameliorated the diminished levels of GSH and GPx. However, it is important to note the limitations of the TBARS assay such as its non-specific reactivity with MDA as well as the possibility of production of MDA from biological reactions unrelated to lipid peroxidation. These results indicate a strong antioxidant action of rosuvastatin which can play an important role in the treatment of the cognitive impairment.

Mitochondrial dysfunction is commonly considered to be one of the culprits that lead to cognitive impairment and various neurological disorders such as Alzheimer’s disease [61]. Determination of MMP was done to assess mitochondrial function and also to evaluate oxidative stress. A decrease in MMP is normally observed in mitochondrial dysfunction due to the reduction in the translocation of hydrogen from mitochondrial matrix to the inter-membrane space. The decrease in MMP induced by HSCD was reversed by rosuvastatin administration. The amelioration of reduced MMP was non-significant in case of RSV5 group, but significant improvements were observed in the RSV10 and RSV15 groups. These results suggested that rosuvastatin has potential role to improve in mitochondrial dysfunction which is associated with HSCD.

In the present study, HSCD increased Nrf2 protein (Figure 7) staining in cortex and CA1 region of the hippocampus as compared to NC group animals. According to these immunohistochemical analysis results, it could be postulated that Nrf2 pathway was activated in brain by HSCD. These results suggested that Nrf2 pathway might play a protective role in the pathological processes induced by HSCD. Nrf2 protein expression was increased by rosuvastatin. Western blot results showed that, when compared to the normal control group, administration of HSCD led to translocation of Nrf2 in the nucleus. Furthermore, treatment with rosuvastatin led to a significant increase in the presence of Nrf2 in the nucleus than in the cytoplasm. These immunohistochemical analysis and western blot results also support the role of rosuvastatin against HSCD-induced cognitive impairment in rats via activation Nrf2–ARE pathway.

One of the hallmarks of apoptotic cell death is the degradation of nuclear DNA into oligonucleosomal units [62]. DNA nicking resulting from exposure to HSCD was detected using a TUNEL assay, in which the proportion of DNA nicks was quantified by measuring the binding of dUTP–FLUOS to the nicked end via TdT. Since, HSCD had been shown to induce apoptosis in rats by disrupting and causing DNA fragmentation including generation of nicked DNA and cell cycle arrest [7]. In addition, caspase 3 activity was significantly increased in animals administered HSCD indicating activation of apoptotic pathway. Treatment with rosuvastatin led to significant decrease in caspase 3 levels and the most pronounced effect was observed with the 15 mg dose of rosuvastatin. Therefore, we can conclude that RSV has potential to reduce apoptosis in HSFD fed rats as demonstrated by the results of TUNEL and caspase 3 assays. Considering the promising nature of the aforementioned observations, we believe that more emphasis should be placed on the Nrf2–ARE pathway and its role in the pathophysiology and treatment of cognitive impairment and dementia.

## Conclusion

On the basis of our results we may conclude that HSCD was significantly associated with induction of cognitive impairment in rats, and that treatment with enzyme HMG-CoA reductase inhibitor, rosuvastatin, ameliorated cognitive impairment via its action on the Nrf2–ARE pathway. The promising nature of our findings encourages further research to investigate the potential of rosuvastatin for the treatment of cognitive impairment.
